# Quantum Simulation of the Shortcut to the Adiabatic Passage Using Nuclear Magnetic Resonance

**DOI:** 10.3390/e25071020

**Published:** 2023-07-04

**Authors:** Xin-Chang Liu, Xiang-Yu Kong

**Affiliations:** 1 School of Electronics Engineering, Beijing University of Posts and Telecommunications, Beijing 100876, China; liuxinchang@bupt.edu.cn; 2 Department of Physics, Tsinghua University, Beijing 100084, China

**Keywords:** shortcut, adiabatic passage, nuclear magnetic resonance

## Abstract

Quantum adiabatic shortcut technology provides a technique to accelerate the quantum adiabatic process and has been widely used in various fields of quantum information processing. In this work, we proposed a two-level quantum shortcut adiabatic passage model. Then, exploiting the nuclear magnetic resonance, we experimentally simulated the dynamics of quantum shortcut adiabatic passage using the water molecules.

## 1. Introduction

The concept of a quantum shortcut adiabatic (QSTA) passage was first proposed by Chen et al. [1]. It describes a non-adiabatic way to accelerate the quantum adiabatic process, which can produce the same population distribution and target state as the adiabatic process. “Shortcut” means that the adiabatic process can be completed in a relatively short time. The Lewis–Riesenfeld invariant-based inverse engineering method is defined as an effective approach to QSTA that expresses the eigenstates of a Hamiltonian from the specified initial to the final configurations and then constructs, from the invariant, the transient Hamiltonian that connects these boundary configurations [2,3]. In recent decades, the shortcut adiabatic passage has been widely applied in atomic physics and optics. For example, QSTA has been used to generate spin-squeezed states in superconductors [4] and the population inversion in two-level quantum systems [5].

The key technique of QSTA is that the time-dependent interactions that are externally applied to the system usually change slowly to maintain the adiabaticity and control the final state of a quantum system robustly compared to the parameter fluctuations. There are several studies that focus on the implementations and techniques of QSTA. For example, in Ref. [6], Berry presented an approach to quantum driving without transition. Later, in [7], Masuda and Nakamura proposed a quantum system that could exhibit fast-forward adiabatic dynamics. Moreover, QSTA techniques have been used in the state engineering of matter waves [8], and also in the spin manipulation of quantum dots [9].

Nuclear magnetic resonance (NMR) occurs when nuclei in a static magnetic field are disturbed by an oscillating magnetic field; then, the nuclei generate an electromagnetic signal with a frequency depending on the applied magnetic field. NMR is a multifaceted technique that enables the analysis of liquid state and solid matter using high-resolution spectrums. The applications of NMR technology in quantum computing and quantum information have developed rapidly. In 1996, Chuang first demonstrated that the nuclear magnetic resonance of the spins could be used in quantum computation [10]. Later, extensive studies were conducted on NMR quantum computing [11,12,13,14], and it has also been used in various branches of quantum information sciences, such as the manipulation of quantum gates [15], and the design of the Hamiltonian [16,17]. The specific steps of quantum computing using NMR include the following steps: the initialization, the realization of quantum gates, the reading of the final states, and quantum control techniques commonly used in NMR [18,19,20].

Here, in this study, we propose a theoretical model of QSTA using a two-level atomic system. Further, based on the NMR system, the dynamics of the QSTA were experimentally simulated using the water molecules. We found that the experimental results agree with the theory, and we believe the method is useful and could be further applied in the simulation of quantum computation and quantum dynamics using NMR.

## 2. Basic Model of Quantum Shortcut Adiabatic Passage

To present the basic theory of QSTA, we chose a two-level atom as a fundamental example. As the speedup versions of adiabatic passage (RAP), the two-level atomic system is useful in the dynamics of chemical reactions, laser cooling [21], and quantum information processing [22,23]. For the two-level atom, the eigenvectors in the Hilbert space could be described by the vectors $|0\rangle ={\left(1,0\right)}^{T},|1\rangle ={\left(0,1\right)}^{T}$. By applying the rotating wave approximation, the time-dependent Hamiltonian in the laser-adapted interaction picture could be expressed as

(1) $$H\left(t\right)=\frac{\hbar }{2}\left(\begin{array}{cc} ▵ & \Omega_{R}e^{i\varphi }\\ \Omega_{R}e^{-i\varphi } & -▵ \end{array}\right),$$

where $▵=▵\left(t\right)$ and $\Omega_{R}=\Omega_{R}\left(t\right)$ are the time-dependent detuning and the Rabi oscillation frequency of the atom, respectively. $\varphi =\varphi \left(t\right)$ denotes the time-dependent phase shift. For simplicity, we assumed φ=0 and only considered the Hamiltonian in the expression as:

(2) $$H\left(t\right)=\frac{\hbar }{2}\left(\begin{array}{cc} ▵ & \Omega_{R}\\ \Omega_{R} & -▵ \end{array}\right).$$

Suppose the invariant operator $I\left(t\right)$ could be expressed as

(3) $$I\left(t\right)=\frac{\hbar }{2}\Omega_{0}\left(\begin{array}{cc} \cos\gamma & \sin\gamma e^{i\beta }\\ \sin\gamma e^{-i\beta } & -\cos\gamma \end{array}\right),$$

where $\Omega_{0}$ is an arbitrary constant, $\beta =\beta \left(t\right)$ and $\gamma =\gamma \left(t\right)$ are auxiliary time-dependent angles. The dynamical invariant $I\left(t\right)$ satisfies the equation

(4) $$i\hbar \frac{\partial I\left(t\right)}{\partial t}-\left[H\left(t\right),I\left(t\right)\right]=0.$$

By substituting the expressions of I(t) and H(t) into Equation (4), we could obtain the relations of the angles and the parameters of the system as

(5) $$\begin{array}{c} \left(\begin{array}{cc} -i\dot{\gamma }\sin\gamma & i\dot{\gamma }\cos\gamma e^{i\beta }-\dot{\beta }\sin\gamma e^{i\beta }\\ i\dot{\gamma }\cos\gamma e^{-i\beta }+\dot{\beta }\sin\gamma e^{-i\beta } & i\dot{\gamma }\sin\gamma \end{array}\right)\\ =\left(\begin{array}{cc} -i\Omega_{R}\sin\gamma \sin\beta & ▵\sin\gamma e^{i\beta }-\Omega_{R}\cos\gamma \\ \Omega_{R}\cos\gamma -▵\sin\gamma e^{-i\beta } & i\Omega_{R}\sin\gamma \sin\beta \end{array}\right) \end{array}$$

The relationship between the variables can be extracted from the above equation as

(6) $$\begin{array}{ccc} \dot{\gamma }\sin\gamma & = & \Omega_{R}\sin\gamma \sin\beta , \end{array}$$

(7) $$\begin{array}{ccc} ▵\sin\gamma e^{i\beta }-\Omega_{R}\cos\gamma & = & i\dot{\gamma }\cos\gamma e^{i\beta }-\dot{\beta }\sin\gamma e^{i\beta }. \end{array}$$

We could simplify the first condition as $\dot{\gamma }=\Omega_{R}\sin\beta$ with sinγ≠0. The detuning could be solved as $▵=\Omega_{R}\cot\gamma \cos\beta -\dot{\beta }$ with sinγ≠0. Since sinγ≠0 is always required in the open range $\left(0,t_{f}\right)$, at two ending times, we will choose γ=νπ in the following.

In order to guarantee that the final state is in the instant eigenstate of $H\left(t\right)$ at the ending time $t_{f}$, the conditions that $\left[H\left(0\right),I\left(0\right)\right]=0$ and $\left[H\left(t_{f}\right),I\left(t_{f}\right)\right]=0$ should be satisfied. Based on these constraints, the boundary conditions at t=0 could be described as follows:

$$\begin{array}{ccc} \Omega_{R}\left(0\right)\sin\gamma \left(0\right)\sin\beta \left(0\right) & = & 0,\\ ▵\left(0\right)\sin\gamma \left(0\right)e^{i\beta \left(0\right)}-\Omega_{R}\left(0\right)\cos\gamma \left(0\right) & = & 0,\\ \Omega_{R}\left(0\right)\cos\gamma \left(0\right)-▵\left(0\right)\sin\gamma \left(0\right)e^{-i\beta \left(0\right)} & = & 0, \end{array}$$

and the condition $t=t_{f}$ could similarly be expressed as:

$$\begin{array}{ccc} \Omega_{R}\left(t_{f}\right)\sin\gamma \left(t_{f}\right)\sin\beta \left(t_{f}\right) & = & 0,\\ ▵\left(t_{f}\right)\sin\gamma \left(t_{f}\right)e^{i\beta \left(t_{f}\right)}-\Omega_{R}\left(t_{f}\right)\cos\gamma \left(t_{f}\right) & = & 0,\\ \Omega_{R}\left(t_{f}\right)\cos\gamma \left(t_{f}\right)-▵\left(t_{f}\right)\sin\gamma \left(t_{f}\right)e^{-i\beta \left(t_{f}\right)} & = & 0. \end{array}$$

By choosing the relations $\Omega_{R}\left(0\right)=0,\gamma \left(0\right)=\pi$ and $\Omega_{R}\left(t_{f}\right)=0,\gamma \left(t_{f}\right)=0$, where $\beta \left(0\right)$ and $\beta \left(t_{f}\right)$ can be chosen arbitrarily, we can obtain

(8) $$\dot{\gamma }\left(0\right)=\dot{\gamma }\left(t_{f}\right)=0.$$

As mentioned before, we can choose the proper values of $\beta \left(0\right)$ and $\beta \left(t_{f}\right)$. According to the above relations derived from Equation (5), we can estimate the optimization parameters in advance. Firstly, β should be kept close to $\left(n+1/2\right)\pi$, as only the results of trigonometric functions affect the solution. To minimize $\Omega_{R}$ along the path, the derivatives would fix the initial and final detunings, which should have opposite signs here. Moreover, the parameter should not be too large to keep β close to the chosen reference value and to avoid β=0 at some intermediate time. Considering all these constraints, we imposed the following relations

(9) $$\begin{array}{ccc} \beta \left(0\right) & = & -\pi /2,\dot{\beta }\left(0\right)=3\pi /\left(2t_{f}\right), \end{array}$$

(10) $$\begin{array}{ccc} \beta \left(t_{f}\right) & = & -\pi /2,\dot{\beta }\left(t_{f}\right)=-3\pi /\left(2t_{f}\right), \end{array}$$

where the negative sign of β keeps $\Omega_{R}$ positive.

Here, we assumed a polynomial ansatz as $\gamma \left(t\right)=\sum_{j=0}^{3}a_{j}t^{j}$ and $\beta \left(t\right)=\sum_{j=0}^{3}b_{j}t^{j}$, where the coefficients could be found by solving the equation set according to the boundary conditions. Analytically, the two parameters γ and β could be solved and expressed as

(11) $$\gamma \left(t\right)=\pi -3\pi t^{2}+2\pi t^{3}.$$

and

(12) $$\beta \left(t\right)=-\pi /2+\left(3\pi /2\right)t-\left(3\pi /2\right)t^{2}.$$

Then, the corresponding functions of $\Omega_{R}$ and Δ could be expressed as:

(13) $$\Omega_{R}=\dot{\gamma }/sin\beta =\left(-6\pi t+6\pi t^{2}\right)/sin\beta$$

and

(14) $$\Delta =\Omega_{R}cot\gamma cos\beta -\dot{\beta }=\left(-6\pi t+6\pi t^{2}\right)cot\gamma cos\beta -\left(3\pi /2-3\pi t\right)$$

**Figure 1:**
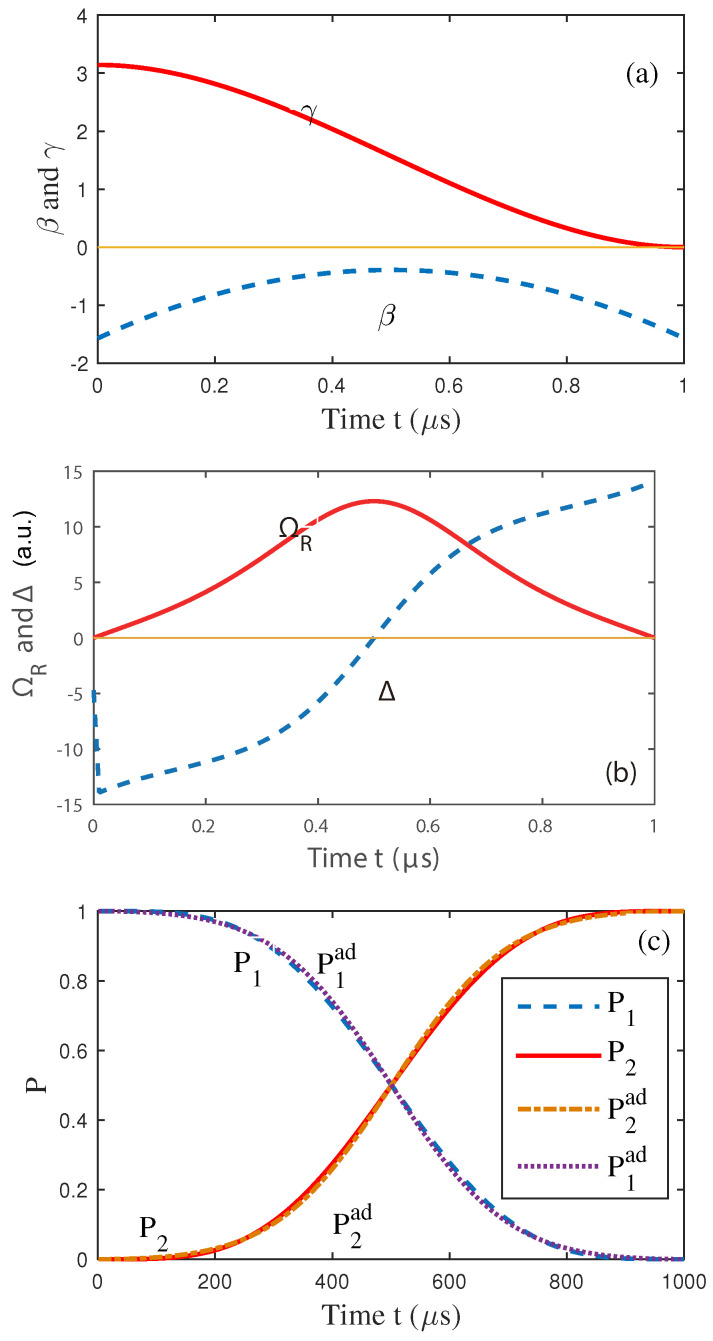
(Color online) (**a**) The evolution of polynomial ansatzes for γ(t) and β(t). (**b**) The evolution of the corresponding functions of $\Omega_{R}$ and Δ. (**c**) Time evolution of the populations of levels 1 and 2. Here, $P_{1(2)}$ denotes the probability of the level-1 (2) during evolution using the shortcut method, and $P_{1(2)}^{ad}$ represents the results using the traditional adiabatic method.

Here, we numerically simulated the evolution of the parameters β, γ, $\Omega_{R}$, Δ in Figure 1a,b, and the population probabilities of the level-states in Figure 1c. Figure 1a denotes the evolution of γ and β along with the time [24]. In Figure 1c, $P_{1}$ and $P_{2}$ represent the probabilities of the two levels, respectively. $P^{ad}$ denotes the probability numerically calculated using the adiabatic method. We can conclude that there is no large difference between this and the previous adiabatic method. The designed protocol is an adiabatic passage for the specified final time $t_{f}$.

**Figure 2:**
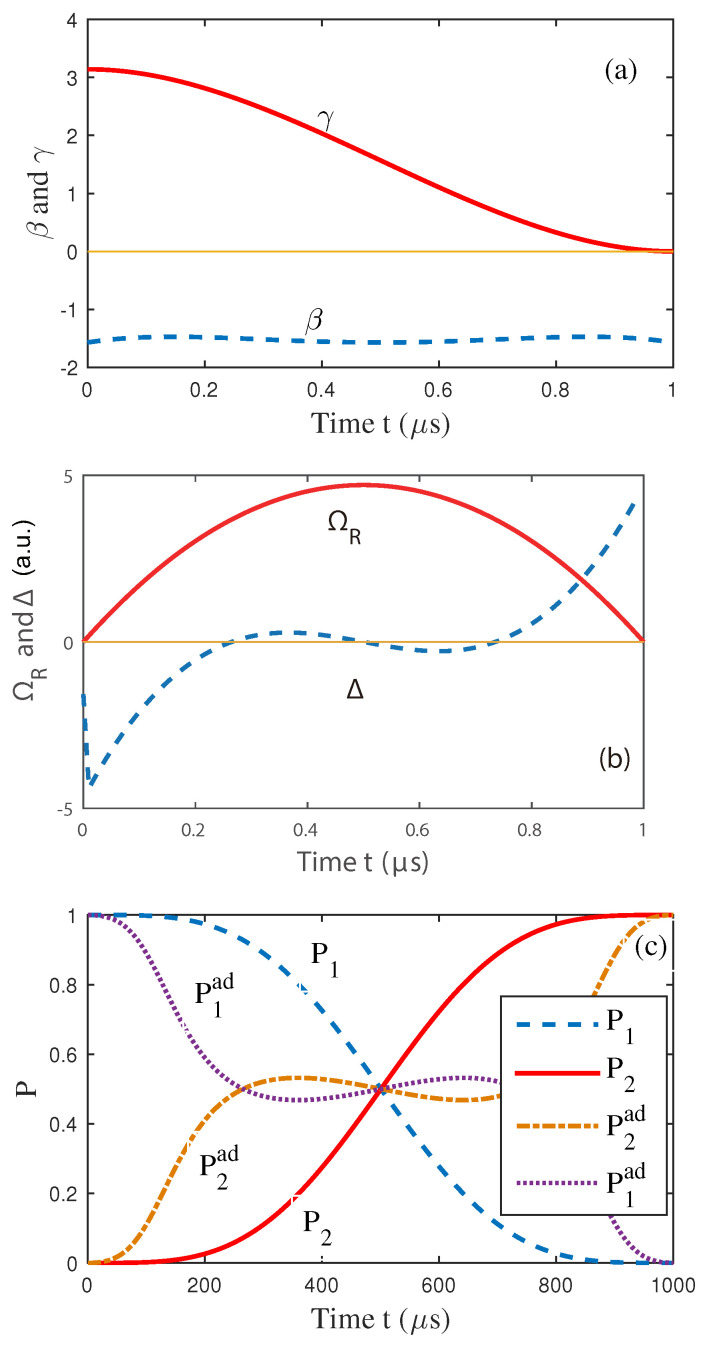
(Color online) (**a**) The evolution of the polynomial ansatzes for γ(t) and β(t). (**b**) The evolution of the corresponding functions of $\Omega_{R}$ and Δ. (**c**) Time evolution of the populations of levels 1 and 2. Here, $P_{1(2)}$ denotes the probability of the level-1(2) during evolution using shortcut method, and $P_{1(2)}^{ad}$ represents the traditional adiabatic method.

Moreover, we imposed some additional conditions at an intermediate time as:

(15) $$\begin{array}{ccc} \beta \left(0\right) & = & -\pi /2,\beta \left(t_{f}\right)=-\pi /2,\beta \left(t_{f}/2\right)=-\pi /2, \end{array}$$

(16) $$\begin{array}{ccc} \dot{\beta }\left(0\right) & = & \pi /\left(2t_{f}\right),\dot{\beta }\left(t_{f}\right)=-\pi /\left(2t_{f}\right), \end{array}$$

to keep β closer to −π/2, where we also diminished the detuning. This new set of conditions requires a higher-order polynomial as $\beta \left(t\right)=\sum_{j=0}^{4}b_{j}t^{j}$. γ was also chosen as the same value as before. The results are presented in Figure 2.

For Figure 2a,b, the evolution of the parameters β and Δ is different from Figure 1. We also found the final results show a relative difference for the adiabatic method, which means that the method can be further complemented by optimizing the trajectory concerning the different physical cost functions or constraints.

## 3. The Quantum Simulation of Shortcut to the Adiabatic Passage Using Nuclear Magnetic Resonance

As mentioned in the above section, we used the nuclear magnetic resonance (NMR) system to simulate the QSTA process. The sample was water with 90% $H_{2}O$ and 10% $D_{2}O$, and the signal of $H_{2}O$ was of interest. The two ${}^{1}H$ spins in the $H_{2}O$ molecule were identical, with the same chemical environment, so it was a single qubit sample, which is what the experiment needed. All experiments were carried out on a Bruker ADVANCE III 400 MHz spectrometer.

The experimental process of the single qubit can be simplified compared with those with more qubits. To begin with, the internal Hamiltonian is zero in the resonant rotating frame. The thermal equilibrium state is the pseudo-pure state (PPS) from which we started our experiment. This can be seen from Equation

(17) $$\sigma_{z}=200-I,$$

where $\sigma_{z}$ is the Pauli Z operator proportional to the thermal equilibrium state and *I* represents the 2×2 identity operator.

Then, we added the radio-frequency (RF) signals on the X-Y plane. Here, the Hamiltonian could be decomposed into a sequence of RF pulses. As the Hamiltonian to be simulated is time-dependent, we used a piecewise constant Hamiltonian with small jumps between steps instead. We set the evolution time to 10 ms and divided the total procedure into 1000 steps (each step is 10 μs), as follows:

(18) $$U=\Pi_{i}^{1000}U_{i}=U_{1000}\ldots U_{i}\ldots U_{2}U_{1}.$$

(19) $$U_{i}=e^{-iH(t_{i})\Delta t}.$$

where Δt=10 μs, $t_{i}=i\Delta t$ and $H\left(t_{i}\right)=\Delta \left(t_{i}\right)\sigma_{z}+\Omega \left(t_{i}\right)\sigma_{x}$ as introduced in Equation (2).

**Figure 3:**
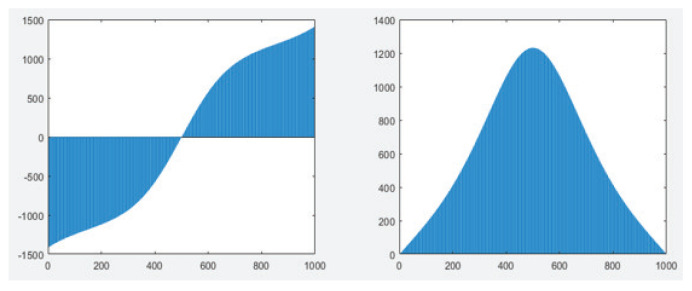
(Color online) RF pulses used in the experiment. The **left** and **right** figures are the RF fields applied in the *x* and *y* directions. The *x*-axis represents evolution time, and the *y*-axis denotes the amplitude of RF pulses.

Moreover, as shown in Figure 3, the RF pulses in the NMR system were applied in the *x*- and *y*-direction, respectively. Thus, we could experimentally realize the Hamiltonian as follows:

(20) $$H^{\prime }\left(t_{i}\right)=\Delta \left(t_{i}\right)\sigma_{y}+\Omega \left(t_{i}\right)\sigma_{x}.$$

In addition, it is obvious that

(21) $$e^{-iH(t_{i})\Delta t}=Xe^{-iH^{\prime }\left(t_{i}\right)\Delta t}X^{†},$$

where *X* denotes a 90-degree rotation around the x direction. Therefore, we have

(22) $$U=XU^{\prime }X^{†}.$$

(23) $$U^{\prime }=\Pi_{i}^{1000}U_{i}^{\prime }.$$

where $U_{i}^{\prime }=e^{-iH^{\prime }\left(t_{i}\right)\Delta t}$, which can be realized practically by *x*- and *y*-direction RF pulses, as shown in Figure 3.

**Figure 4:**
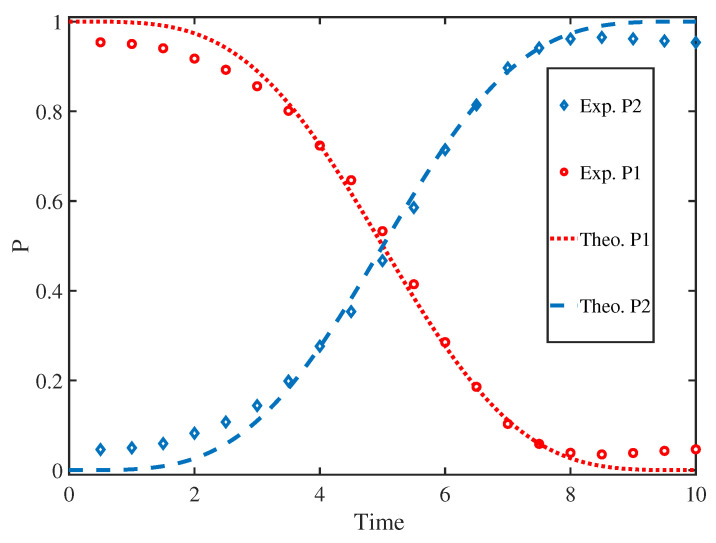
(Color online) The experimenta; results of the simulation. Here, the red dotted line and the blue dashed line represent the theoretical population of 0 and 1, respectively. The red circles and blue rhombus are the experimental populations of 0 and 1 at each step, respectively.

By fitting the spectrum, we measured the population of |0> in the final state. Then, by performing the experiment after 50 steps, the results were obtained and are shown in Figure 4. Here, $P_{1}$ and $P_{2}$ represent the population of the two levels, respectively. The red circles denote the experiment result of the |0> state population, and the blue rhombus represents the experiment result of the |1> state population. These results are fitted by the red dotted line and the blue dashed line, respectively. Then, we compared the theoretical results with the experimental results of both $P_{1}$ and $P_{2}$; it is obvious that the experiment results show a good agreement with the theory. In this figure, the horizontal coordinate represents the evolution time of the population, and the vertical coordinate represents the population of the final state. It should be noted that the time evolution of the Hamiltonian is simulated by the RF pulses, so the evolution process simulated is not strictly along the time scale as only a unit of time with quasi-time parameter.

Meanwhile, we noticed that the loss of the experiment signals would reduce the fidelity of the experiment. The loss of experimental signal is mainly due to the decoherence and inhomogeneity of the radio-frequency (RF) field and pulse imperfection. For the second effect, we considered the external RF pulse signal applied to the sample. Here, the pulses were added to the *x*-axes and *y*-axes, respectively. The in-homogeneous pulses will induce noise on both the amplitude and the phase of the pulses. We theoretically simulated the noise by changing the amplitude and phase values of the pulses by flipping the signs and comparing the density matrix with the theoretical value of the final state. We found that the fidelity of the theoretical simulation results was greater than 0.99, which means the current scheme is robust to RF signal in-homogeneity. These will affect the state of nuclear spin in NMR experiments. According to the theoretical simulation, the fidelity of the state could be kept larger than 0.99, which means that the experimental results are robust to the loss and decoherence effect. We believe the method developed in this experiment for quantum computation using the NMR system can also be extended to a multi-qubit system.

## 4. Summary

In summary, we present a theoretical model that was used to study the dynamics of QSTA and experimentally simulate the process using NMR systems. The experimental results show a similar effect to the theory. The NMR quantum computing platform has several advantages, such as the long coherence time, robustness, and ease of reading the results. This makes it a promising platform for quantum computing and quantum simulation. Although the formulation of the method is general, explicit constructions of the Hamiltonian are restricted to simple systems such as two- and three-systems, harmonic oscillators, and scale-invariant systems. We believe that the current study could provide a new approach to further investigate NMR systems in quantum information sciences.

## Data Availability

Not applicable.
